# PBF-EB of Fe-Cr-V Alloy for Wear Applications

**DOI:** 10.3390/ma15051679

**Published:** 2022-02-23

**Authors:** Marie Franke-Jurisch, Markus Mirz, Thomas Wenz, Alexander Kirchner, Burghardt Klöden, Thomas Weißgärber

**Affiliations:** 1 Fraunhofer Institute for Manufacturing Technology and Advanced Materials IFAM, 01277 Dresden, Germany; alexander.kirchner@ifam-dd.fraunhofer.de (A.K.); burghardt.kloeden@ifam-dd.fraunhofer.de (B.K.); thomas.weissgaerber@ifam-dd.fraunhofer.de (T.W.); 2 Institute for Materials Applications in Mechanical Engineering, RWTH Aachen University, 52062 Aachen, Germany; m.mirz@iwm.rwth-aachen.de; 3 Herau Anlagentechnik GmbH, 44289 Dortmund, Germany; t.wenz@herau.de

**Keywords:** PBF-EB, electron beam melting, additive manufacturing, tool steel, microstructure, carbides, mechanical properties, wear, recycling industry

## Abstract

Due to the small variety of materials, the areas of application of additive manufacturing in the toolmaking industry are currently still limited. In order to overcome these material restrictions, AM material development for high carbon-containing iron-based materials, which are characterized by high strength, hardness, and wear resistance, must be intensified. However, these materials are often susceptible to crack formation or lack of fusion defects during processing. Therefore, these materials are preferentially suited for electron beam powder bed fusion (PBF-EB). In this paper, an Fe-Cr-V alloy with 10% vanadium is presented. Investigations were carried out on the PBF-EB system Arcam A2X. Specimens and demonstrators are characterized by a three-phase microstructure with an Fe-rich matrix and VC and M_7_C_3_ reinforcements. The resulting microstructures were characterized by scanning electron microscopy (SEM) and electron backscatter diffraction (EBSD). Furthermore, mechanical and physical properties were measured. A final field test was conducted to evaluate durability in use.

## 1. Introduction

Additive manufacturing (AM), especially powder bed fusion (PBF) technologies, such as laser beam powder bed fusion (PBF-LB) and electron beam powder bed fusion (PBF-EB), have been proven suitable for the production of complex-shaped parts. However, most of the materials that have been investigated were non-iron based, as their manufacturing was often combined with high production and material costs, or stainless or maraging steel [1,2,3,4]. Interest in the use of AM for cost-driven industries, tool steel applications in particular, has been increasing recently, which is reflected in the number of publications in the last years [5,6,7,8,9,10,11,12,13,14,15,16,17,18,19,20,21,22,23,24,25].

However, there is still a lack of investigations dealing with AM of highly wear-resistant carbon containing tool steels. The reason lies in the challenges that arise during rapid solidification for PBF-LB especially, as high residual stresses often lead to crack formation. Most common publications from Sander, Botero, etc. deal with an FeCrMoVC alloy, Uddeholms Vanadis 4 Extra©, Vibenite© 480 by VBN Components, FeCu-C alloy, and FeCr-10V, respectively [7,10,11,23,25,26].

Applications that are addressed within this study are cutting tools such as cutting crowns or knifes for shredding processes, which typically consist of wear resistant tool steels such as 1.2379. Currently, they need to be replaced regularly because they wear out quickly. This leads to down times on the recycling machines as well as increased personnel and material costs during replacement. Materials with higher wear resistance would be desirable, but so far, they can only be applied cost-effectively as thin hard facings with better, but still with limited service life. The state of the art is the coating by laser cladding, arc welding, or thermal spraying such as plasma transferred arc welding (PTA) [27]. The reason for the limited thicknesses is the high residual stresses that arise during deposition. Additionally, with higher energy inputs, dilution effects may occur reducing the wear resistance [27,28]. If those materials could be produced that are thicker, with a longer lifetime, then lower costs would be expected. Here, PBF-EB has proven suitable as an alternative for the processing of wear resistant parts [25]. Due to its high build temperatures of up to 1000 °C, the thermal gradient, and thus, the residual stresses in the final parts can be reduced resulting in crack-free components.

Materials that are often discussed for wear and impact applications are powder metallurgical (PM) Fe-Cr-C steels, whereas vanadium carbide (VC) additions are favorable as they raise the wear resistance and lead to a fine-grained Fe-based matrix with homogeneously distributed vanadium and chromium carbides [27,29,30].

This investigation focuses on the wear behavior and applications of the cold work tool steel FeCr-10V processed by PBF-EB. Therefore, cutting crowns with a size of 40 mm × 40 mm × 20 mm as well as cylinders, and other thin-walled samples and bars were printed for further analyses. Microstructural investigations on grains and carbide formation were studied using electron backscatter diffraction and X-ray diffraction. Further examination of key physical properties and application-oriented mechanical and wear properties on the PBF-EB processed FeCr-10V are presented for the first time. Finally, the results of a field test of heat-treated PBF-EB cutting crowns in a post-shredding machine under real-world conditions is discussed.

## 2. Materials and Methods

### 2.1. PBF-EB Processing

The powder material for the PBF-EB trials was a cold work tool steel (FeCr-10V) with the main alloying elements vanadium (10 wt.%), chromium (7 wt.%), and carbon (2.4 wt.%). The gas atomized powders were obtained from m4p material solutions GmbH, Germany with a powder fraction of 50–150 µm. Corresponding flowability of 16 s/50 g and a tapped density of 4.8 g/cm³ were sufficient for the printing of parts with at least 99.8% of the theoretical density. A detailed powder analysis is reported in a previous publication [25].

**Figure 1:**
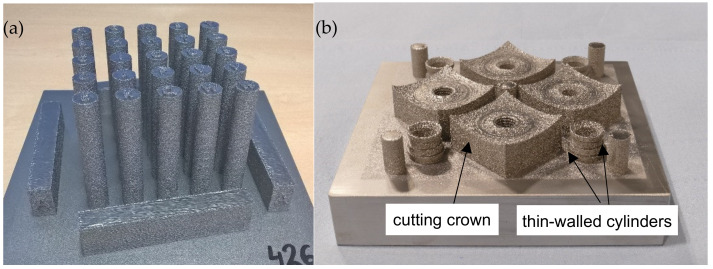
(**a**) PBF-EB printed specimens for mechanical and physical tests; (**b**) demonstrators (cutting crowns and thin-walled open and closed cylinders).

All experiments were prepared on an Arcam A2X machine with an accelerating voltage of 60 kV on a tool steel-based start plate with a build size of 150 × 150 mm^2^. Three different build jobs (see Figure 1) were conducted with a cross snake scan strategy and a layer thickness of 70 μm, while the build temperature was 850 °C. Melting was performed with a line offset of 50 µm and an area energy of about 4 J/mm^2^. Printed cylinders and bars (Figure 1a) were used for mechanical and physical tests, whereas cutting crowns (Figure 1b) served as demonstrators and samples for the field test. A third build job was used for the impact tests including cuboids with a size of 10 mm × 48 mm × 48 mm. The closed cylinders (Figure 1b) are based on thin-walled samples with entrapped powder that were used for density measurements. The wall thicknesses varied from 0.5 to 2 mm. The other cylinders are open on one side with a wall thickness of about 2 mm. Finally, four cutting crowns could be printed this way. The latter build job was repeated twice in order to have enough cutting crowns for the field test.

### 2.2. Heat Treatment

Heat treatment was carried out to increase the wear performance of the cutting crowns and to homogenize their as-built microstructures. Additionally, the selected high hardening temperatures from preliminary studies [25] are not recommended for other tool steels such as H11 or H13 [31,32]. Therefore, a lower austenitizing temperature was chosen to allow balanced hardening even in hybrid production, for example, by printing on a tool steel semi-finished product. The objectives for hardness were only 53–58 HRC. The target parameters are shown in the following table (Table 1). Thereby, the samples were austenitized at 10 K/min up to 1020 °C and kept at this temperature for 60 min. Subsequently, the samples were quenched in oil up to approximately 500 °C, and then, cooled in air to room temperature. The final annealing was carried out three times at 540 °C for 60 min each.

### 2.3. Characterization

For analysis of the micrographs, the specimens were cut, embedded, and polished. Microstructure and phases were examined by scanning electron microscopy (SEM) on a Zeiss EVO 50 XVP and electron backscatter diffraction (EBSD) as well as energy-dispersive X-ray spectroscopy (EDX) on a Helios Nano Lab Dual Beam (Thermo Fisher Scientific, Waltham, MA, USA) with TSL OIM Data collection 7 (EDAX, Ametek, Berwyn, PA, USA). The crystalline phases were double-checked by X-ray diffraction (XRD) on a Bruker D8 Advanced using Cu as the emitting metal and a step-size of 0.01° with a holding time of 4 s.

Density measurements were conducted on printed closed cylinders with entrapped powder to check the density of different wall thicknesses by gas pycnometry, using a AccuPyc 1330 (Micromeritics Instrument Corp., Norcross, GA, USA). 

Additionally, physical properties of the FeCr-10V were investigated. Thermal conductivity was measured on a Netzsch LFA427 Laserflash. Differential scanning calorimetry (DSC) measurements were carried out for determining the heat capacity on a Netzsch DSC 404 Pegasus and thermal expansion was studied by means of dilatometry on a Netzsch DIL402C.

Mechanical and wear properties were determined by hardness, three-point bending, and impact toughness measurements, as well as jet wear testing. Hardness was measured on an Innovatest Falcon 500 (HV10) (Innovatest GmbH, Selfkant-Heilder, Germany), according to DIN EN ISO 6507-1. The three-point bending test according to DIN EN 843-1 was performed on a InspektTable 100 from Hegewald & Peschke at room temperature. Specimens with a sample size of 3 × 4 × 45 mm were cut from blanks using electrical discharge machining. Wear and impact tests were investigated by Herau Anlagentechnik GmbH (Dortmund, Germany) on its own test rigs, as described in the following. 

#### 2.3.1. Jet Wear Test

**Figure 2:**
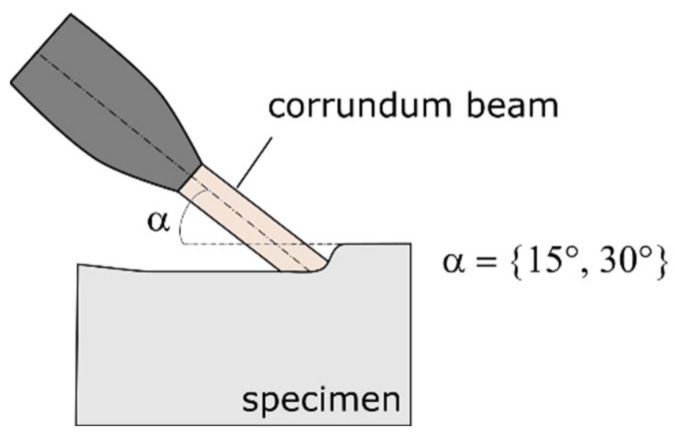
Schematic setup of the jet wear test for hard facings.

Wear can occur in a wide variety of ways, with different wear mechanisms coming into play. For this reason, not all wear stresses can be checked equally by standardized tests such as the pin-on-disc test after ASTM G99 [33]. The latter test cannot reflect the real wear in shredding machines. In other words, good results in terms of low mass loss do not automatically mean the same in practical tests. Experience has shown that a blast wear test with different jet angles is more suitable to compare protection coatings and is carried out by Herau Anlagentechnik GmbH in the following way (see Figure 2). The jet wear tests are used by Herau to compare wear protection coatings for shredding applications. The sample surfaces are blasted with a corundum beam at different angles and the mass removal is measured (Figure 2). In the present measurements, a high-grade corundum of grit size F100 (106–150 μm) was used. The values of the removal rate are normalized to a reference value and presented in a reciprocal manner as a resistance factor. A PTA-welded Fe-Cr-V specimen (FeV12 PTA) was used here as a reference layer.

#### 2.3.2. Impact Test

**Figure 3:**
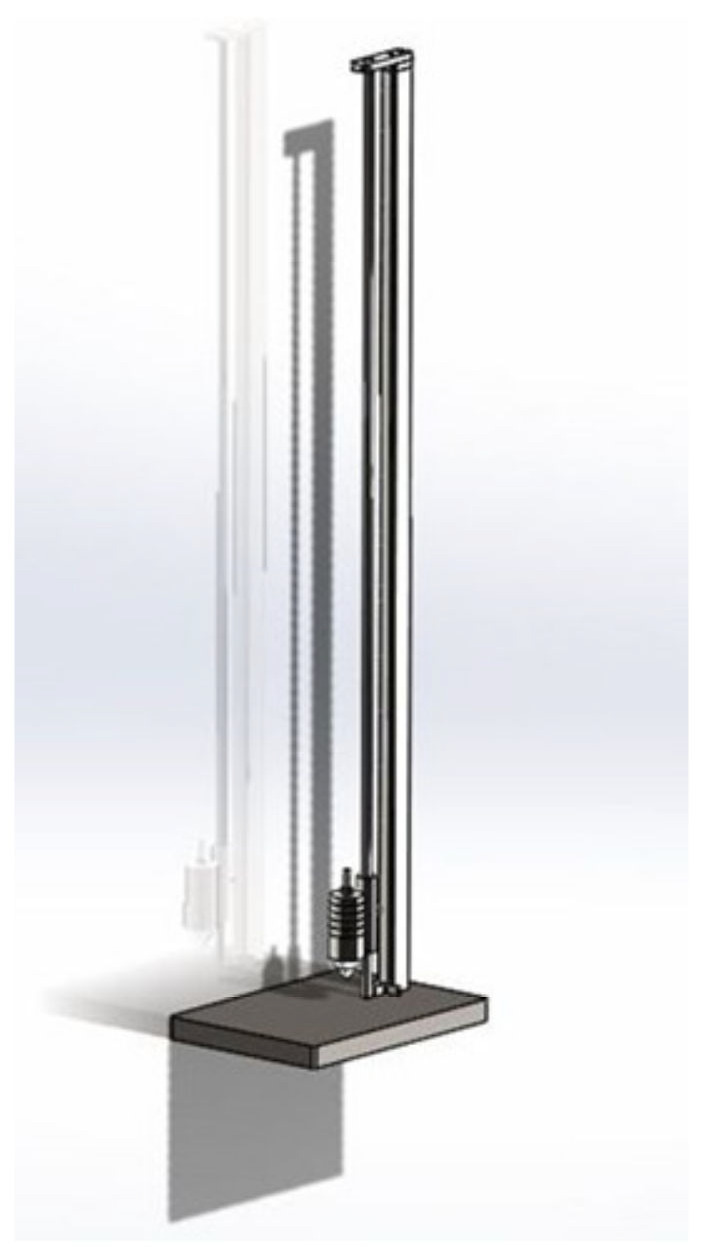
Schematic setup of the test rig for impact toughness measurements.

The impact test was investigated by a drop weight test on both the as-built and the heat-treated samples, and then compared to a reference material (FeV12 PTA and quenched and tempered 1.2379). For these comparative studies, Herau Anlagentechnik GmbH uses a test rig in which a weight falls onto the component from a variable vertical distance (see Figure 3). The test weight has a conical shape, which is fitted with a hardened steel ball (d = 7 mm) on its bottom. The ball hits the specimen at an adjustable distance from the outer edge of the sample and transfers the impact energy. Based on the damage, it can be determined from which drop energy the specimen is no longer deformed, but cracks or chipping occur.

#### 2.3.3. Final Field Test

In order to investigate the failure behavior in a real-life environment, a field test was carried out in which the PBF-EB cutting crowns were placed in the various load ranges of a post-shredder (machine VHZ 1100, Vecoplan GmbH, Bad Marienberg, Germany). The shredding medium was bulky waste (wood and furniture). After 41 h and a throughput of 6 t/h, the crowns were removed for characterization. The wear resistance was determined based on the mass removal rate of the cutting tools. For this purpose, the crowns were weighed before and after the field test. The determined resistance factor was calculated from the individual mass loss in relation to the mass loss of the standard (1.2379).

## 3. Results and Discussion

### 3.1. Microstructural Analysis

**Figure 4:**
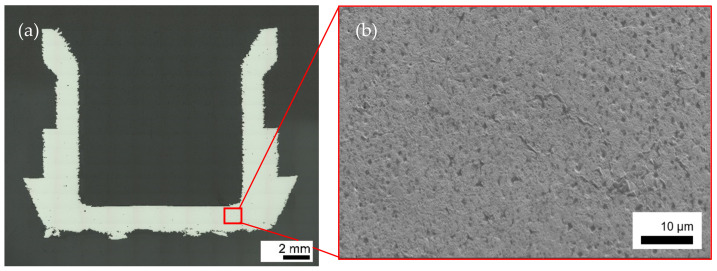
Cross section (**a**) and corresponding SE image (**b**) of the open thin-walled cylinder presenting a nearly dense and crack free as built microstructure.

The cross section of an as built FeCr-10V thin-walled structure is represented by Figure 4a, showing a crack-free and dense sample. Although the wall thickness of 2 mm is much thinner, the microstructure is similar to previous investigations on 10 mm cubic specimens or bigger (Figure 4b) [25]. 

**Figure 5:**
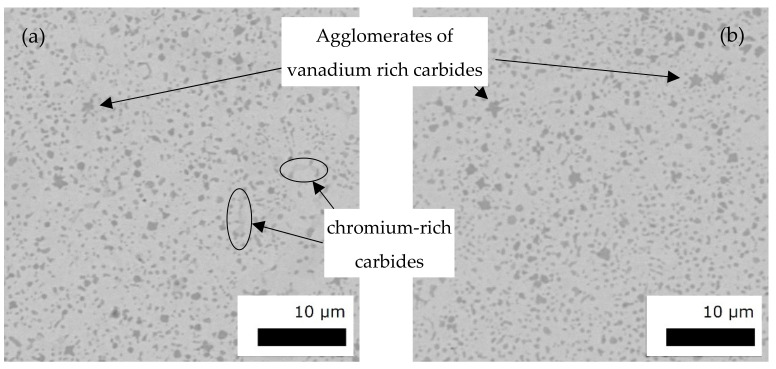
BSD of a cutting crown in as-built state (**a**) and after heat treatment (**b**) presenting a nearly dense and crack free microstructure with a hardness of 570 HV10 and 630 HV10, respectively.

The microstructure of the cutting crowns revealed a nearly dense and crack free microstructure, too, as given by the cross section in Figure 5a. In the as-built state, hardness values were measured to 563 ± 3 HV10 which is already in the range of the target. This is reasoned by the small and rounded vanadium-rich carbides that appear homogeneously distributed in the matrix. The hardness of such single vanadium carbides is about 2800 HV [34]. As these carbides could already be found in the starting powder with similar sizes and are quite stable, they were not fully dissolved during PBF-EB. Consequently, they acted also as a barrier for grain growth in the matrix. Thus, although the material was exposed to high temperatures of at least 850 °C for several hours, the resulting microstructure was very finely grained. 

Similar materials from the literature revealed a slightly different microstructure. In the case of laser cladding carbide formation depended strongly on the beam strategy and composition. As-cladded areas show eutectic and primary carbide formation accompanied by martensitic as well as austenitic structures depending on the composition of the Fe-Cr-V alloys, whereas the microstructure in the reheated zone is similar to PBF-EB ones showing mainly primary carbides embedded in a fine-grained matrix [27,35,36,37]. This leads to the conclusion that the PBF-EB microstructure was more like a heat-treated state. Thus, after heat treatment the microstructure did not change significantly, whereas the hardness could be raised to 630 ± 5 HV10 (see Figure 5b). This is caused by martensite formation and decomposition and the more homogeneous precipitation of chromium carbides. These precipitate so small after heat treatment, that they are no longer visible. The hardness values agree with those found by Wang for CPM-10V for similar ageing temperatures [35].

For both states some of the vanadium-rich primary carbides formed star-shaped agglomerates. Similar observations were made by Günther during gas metal arc welding of hard facings [27]. 

**Figure 6:**
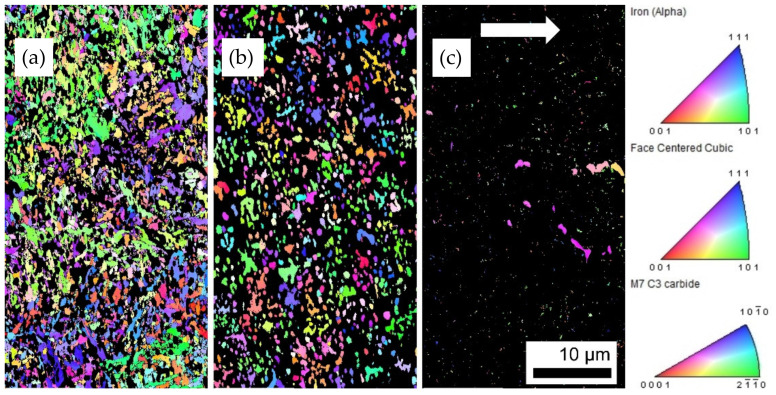
IPF of detected crystalline phases M7C3 (**a**); ferrite (alpha) (**b**); and fcc phase (**c**), in the as-built FeCr-10V open thin-walled cylinder. White arrow indicates the build direction.

**Figure 7:**
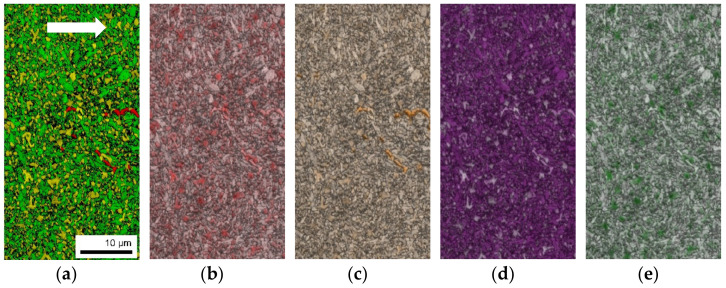
EBSD measurements on the as-built cross section of the open thin-walled cylinder in build direction: (**a**) EBSD phase map; EDX measurements showing the distribution of (**b**) carbon; (**c**) chromium; (**d**) iron; (**e**) vanadium. Build direction is rotated by 90° and indicated by a white arrow.

To further understand the microstructure, EBSD and EDX measurements were conducted on the as-built thin-walled sample from Figure 4, to determine the grain and carbide morphology and sizes, as well as the main phases that were built during solidification. The EBSD inverse pole figures (IPF) demonstrate that there is no significant preferential crystallographic orientation for the main phases, and thus, no texture or orientation along the build direction which might arise in PBF-EB microstructures (see Figure 6). This is mainly reasoned by the in situ heat treatment during PBF-EB and the vanadium-rich carbides that act as obstacles against grain growth [38]. The M_7_C_3_ carbides seem to be distributed inhomogeneously in the matrix both in the case of size and orientation. Figure 6 reveals rod-like M_7_C_3_ carbides of up to 3 µm that, together with the EDX mapping in Figure 7, can be determined to be chromium-rich carbides. They are accompanied by fine dispersed M_7_C_3_ carbides in the matrix.

The corresponding EBSD phase map illustrates the main phase in the microstructure (51.1%) of the PBF-EB FeCr-10V sample as-body centered cubic (bcc) (Figure 7a, green phase). The two other crystallographic structures, face-centered cubic (fcc) (Figure 7a, yellow phase) and hexagonal (hex) M_7_C_3_ type (Figure 7a, red phase) that were detected exhibit a fraction of 25.8% and 2%, respectively (see Table 2). 

**Figure 8:**
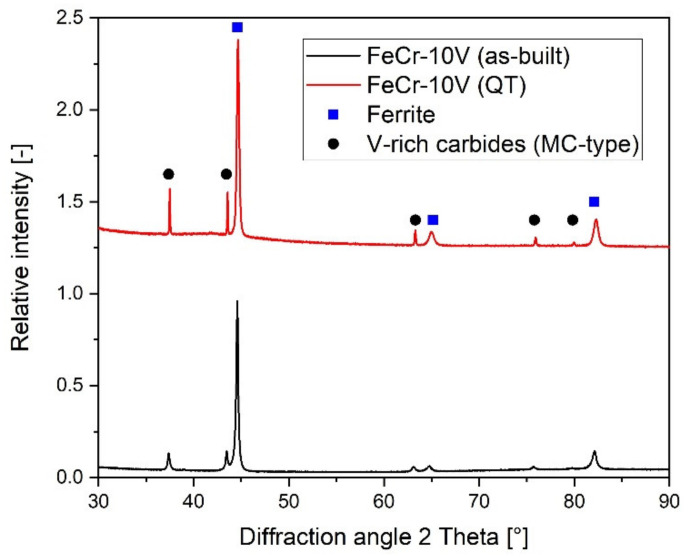
XRD measurements of as-built and heat-treated (QT) FeCr-10V cutting crowns with phases being detected as ferrite (alpha) and fcc (V-rich carbides).

In addition, the XRD measurements were performed on the as-built and the heat-treated conditions, which further determined the microstructural changes (see Figure 8). Samples were cut along the build direction at a 5 mm distance to the edges. The diffractograms revealed similar structures, which agreed with the SEM images in Figure 5. In addition to the main phase of ferrite, only the vanadium-rich carbides in form of ordered stochiometric VC could be detected, which corresponded to the fcc-phase detected by EDX. However, it was still unclear if some of those carbides could also have been ordered V_8_C_7_ which were found by Wang and Leunda et al. [35,36]. V_8_C_7_ crystallizes in the space group P4_3_32 and is only slightly different to the fcc structure of VC [39]. 

The diffractograms additionally confirmed that retained austenite must be very low as it could not be detected by actual XRD measurements. Further analyses with higher resolutions might be able to visualize it, as Leunda and Tekumalla published the occurrence of retained austenite for their CPM 10V or similar material in the as-built state of laser claddings and PTA coatings. Nevertheless, Wang et al. showed that it disappears after heat treatment, confirming the assumption of in situ heat treatment during PBF-EB [35,36,40,41]. Finally, no chromium carbides could be detected by XRD, which was expected due to the small amount of the phase (2 vol%) obtained by EDX (see Table 2).

From calculations on the maximum amount of VC in the FeCr-10V of about 16 vol%, based on the composition from prior publication [25], the detected 25.8 vol% cannot be attributed exclusively to VC. Integrating uncertainties in the composition due to slightly varying chromium and vanadium contents, at least 6 vol% still remain unresolved. They must be associated with retained austenite or other imbalance phases. These considerations lead to the conclusion of an inhomogeneous microstructure, which unfortunately is not visible due to the small area of analysis and the fine microstructure.

The missing approximately 21 vol% from Table 2 could not be verified by EBSD or by XRD measurements. However, they must be a combination of martensite, to a small fraction of other carbides such as M_23_C_6_, and some imbalanced phases, further agreeing with the aforementioned conclusion of an inhomogeneous microstructure. Due to the rapid solidification in the as-built condition, the microstructure is not in equilibrium.

**Figure 9:**
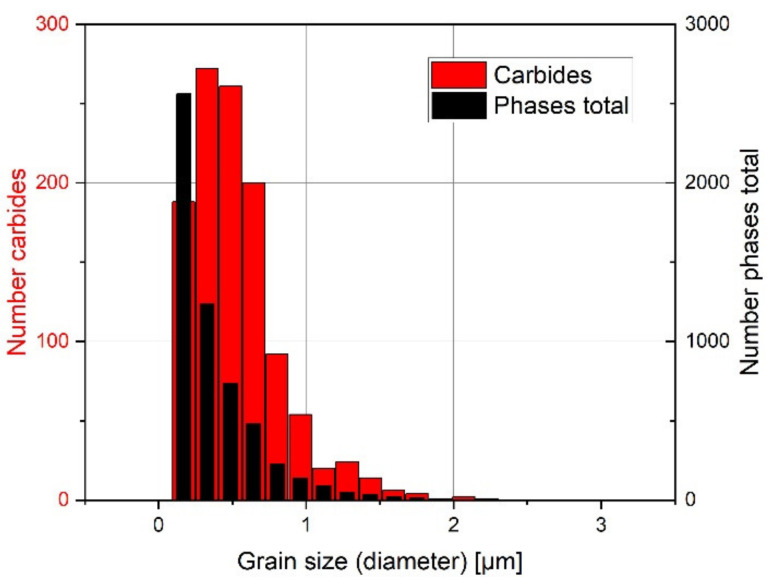
Grain size distribution of all phases in total (black) and carbides (red) from EBSD measurements in Figure 6.

The phase map from Figure 7a gives further information on the grain sizes. Their distribution is shown in Figure 9, wherein the maximum grain size was 3.0 µm, but more than 90% of the grains were smaller than 0.8 µm. The maximum carbide size was 2.2 µm, while 64% of the carbides had a size between 0.3 and 0.6 µm. The high solidification rate resulted in a fine microstructure, with the multiple VCs acting as a barrier to epitaxial grain growth during solidification after melting and remelting. Accordingly, the microstructure exhibited nearly equiaxed fine grains.

### 3.2. Mechanical Properties

#### 3.2.1. Three-Point Bending Test

**Figure 10:**
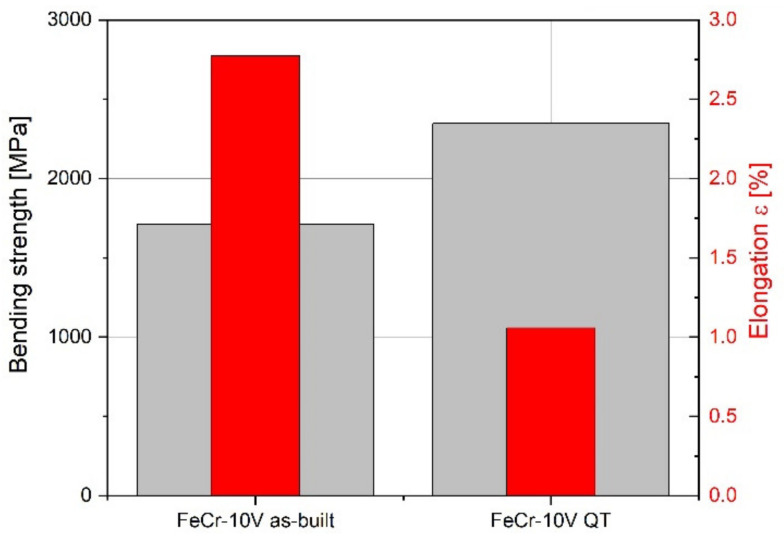
Three-point bending test for the as-built and heat-treated (QT) FeCr-10V PBF-EB mechanical test samples.

For prediction of the strength of brittle materials, the three-point bending test is suitable. The results that are presented in Figure 10 show a significant influence of the heat treatment leading to higher strength but lower elongation. This is reasoned by the martensitic transformation and carbide precipitation during heat treatment. From the values obtained, it can be concluded that further optimization of the heat treatment can lead to a more balanced relationship between strength and ductility, where elongation is between 1 and 2.5% and bending strength is increased as compared with the as-built condition.

#### 3.2.2. Jet Wear Test

**Figure 11:**
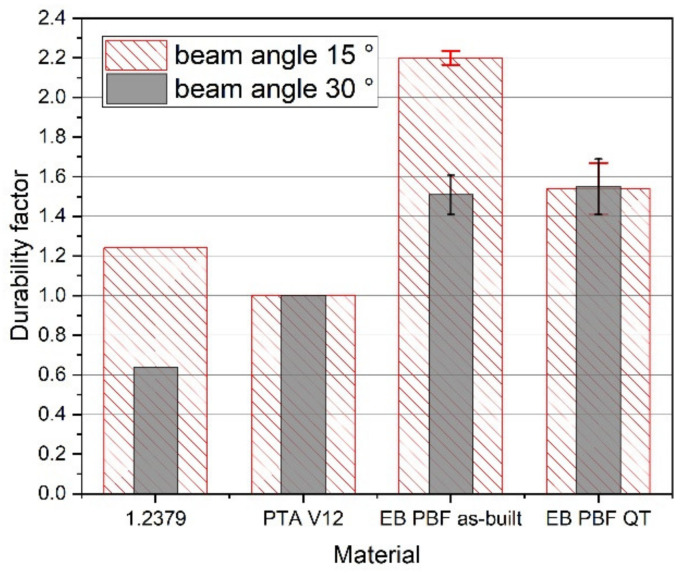
Durability factor dependent on beam angle and material, whereas PTA hard facing is used as reference (factor 1). All PBF-EB samples showed a higher durability.

The durability factors of the PBF-EB specimens were significantly higher than those of the reference coatings (see Figure 11). With a factor greater than 2, the specimens in the as-built state exhibit very good wear resistance. The heat-treated (QT) specimens prove to be more resistant than the reference with a factor of 1.5. The good durability is reasoned by, on the one hand, a thicker wear coating as compared with PTA hard facings, which normally do not exceed a few millimeters [28]. On the other hand, the FeCr-10V produced via PBF-EB and upon slow cooling from build temperature shows a relatively homogeneous microstructure without high residual stresses that might lead to early failure. 

#### 3.2.3. Impact Test

**Figure 12:**
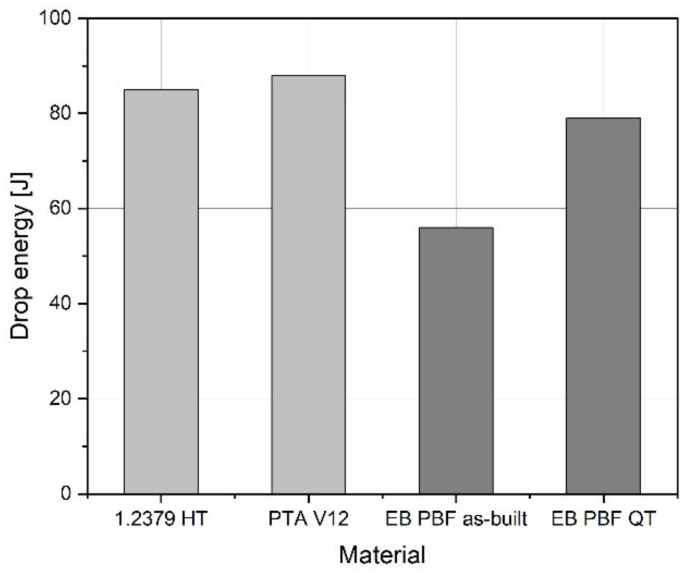
Drop energy dependent on material.

The diagram in Figure 12 represents an overview of drop energies for different materials. The values reflect the condition in which slight cracking may occur but not breakage. The drop weight test revealed lower drop energies for PBF-EB samples as compared with the reference materials, quenched and tempered 1.2379 (cold work tool steel) [42] and the PTA V12 hard facing on conventional tool steel. The reason for the low energy of 56 J for the as-built PBF-EB sample lies in the partly inhomogeneous microstructures. Martensitic areas without ductility could be involved, leading to sudden failure. However, in heat-treated state, the values are similar to the reference, which supports the prior assumption. 

### 3.3. Physical Properties

#### 3.3.1. Density

**Figure 13:**
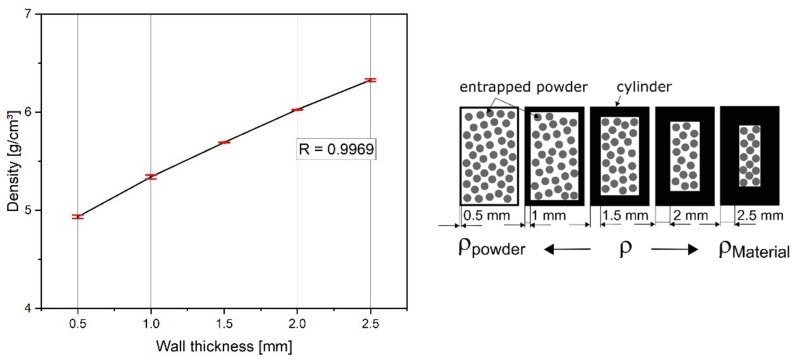
(**Left**) Density of the as-built PBF-EB thin-walled closed cylinder. Reduced density for small wall thicknesses indicates that there is no open porosity in the walls. (**Right**) Density of thick-walled closed cylinders tends to material density.

The density of the cylindrical specimens from Figure 1b was determined as a function of wall thickness to investigate whether closed porosity was still present even with the thin walls. The results are presented in Figure 13 with a high coefficient of determination R of 0.9969. Hereby, a reduced density for small wall thicknesses is preferable and indicates that the walls are nearly dense. As the samples are filled with unmolten powder, the density of the samples must be lower than the material density. Similar to the density measurement of powders, in the case of a porous wall, the density of the material FeCr-10V would be measured instead of the density of the cylinder. Thus, the density would jump to the material density of about 7.4 g/cm³.

For the purpose of completeness, the density of the FeCr-10V solid material was also measured using a gas pycnometer and resulted in 7.2499 g/cm^3^ which was a relative density of 98.7% as compared with the theoretical density. This supports the hypothesis of closed porosity of the thin-walled samples, from Figure 13.

#### 3.3.2. Thermal Conductivity

**Figure 14:**
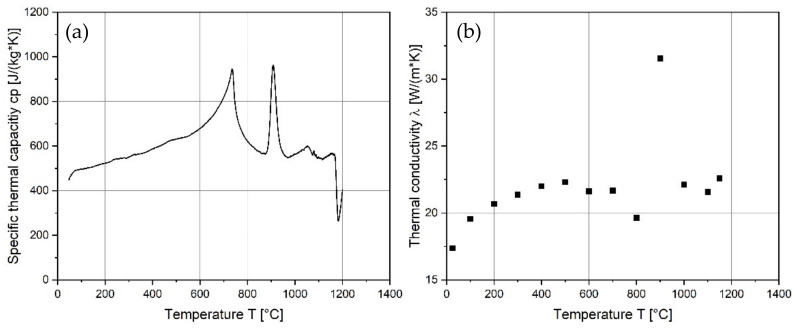
Physical properties of the as-built PBF-EB thin-walled cylinder for tool developments: (**a**) Specific thermal capacity c_p_; (**b**) and conductivity λ.

The evaluation of thermal capacity as well as thermal conductivity and expansion is important in the tool making industry, for designing tools and tool cooling concepts, for avoiding misfits during use under higher temperatures, and for designing heat treatments such as hot isostatic pressing. Measured values are presented in Figure 13 as a function of temperature, respectively. The first peak at about 735 °C is related to the ferromagnetic to paramagnetic transition at the Curie point (Figure 14a). The specific heat measurements reveal a ferrite or martensite to austenite phase transformation with the corresponding peak at about 908 °C. The thermal conductivity of FeCr-10V samples at room temperature is similar to that of alloyed steel, between 15 and 20 W/(m*K) (see Figure 14b) [43].

**Figure 15:**
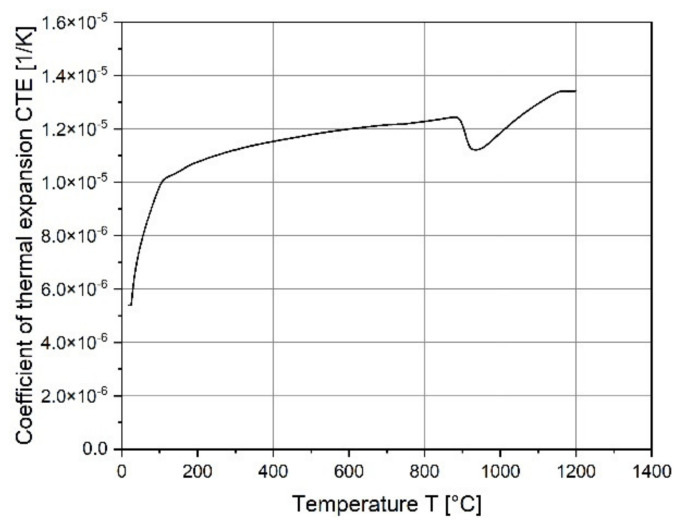
Temperature dependent coefficients of thermal expansion CTE for as-built FeCr-10V.

The coefficients of thermal expansion (CTE) are shown in Figure 15 and are in good agreement with that of a similar tool steel CPM 10V [44]. 

### 3.4. Field Test

**Figure 16:**
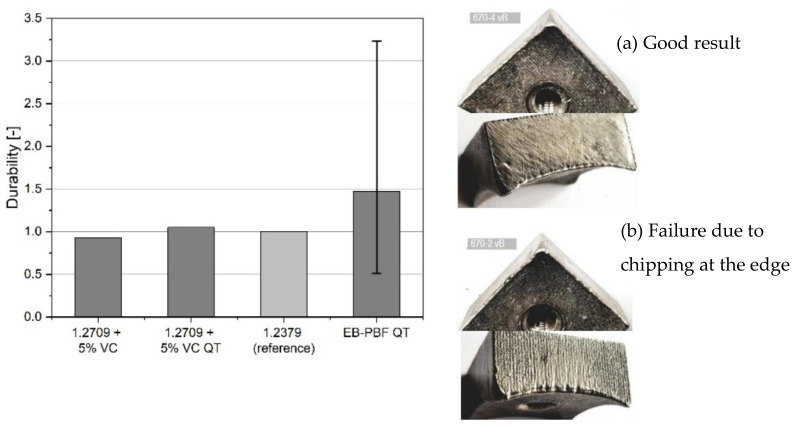
(**Left**) Durability factor dependent on material, whereas conventional 1.2379 is used as reference (factor 1). All PBF-EB samples showed a higher durability. (**Right)** Used cutting crowns with still good shape (**a**) and impact damage (**b**).

The results of the field test are represented in Figure 16 and show the durability of PBF-EB samples as compared with the reference material, conventionally produced 1.2379, and to PBF-LB samples based on 1.2709 with a reinforcement of 5% VC. In general, FeCr-10V PBF-EB samples revealed a very good result with a durability that doubles or even triples the standard material. This means that the mass loss of these specimens is only half or less the mass removal rate of the reference. It is the same for the quenched and tempered VC-reinforced tool steel 1.2709 that already reached the reference values. However, the scatter for PBF-EB is very high. On the one hand, the composition of the waste is reasonable for this, since nails, screws etc. are often unpredictable projectiles that lead to failure even on standard materials. Moreover, there is a lack of data for the PBF-EB samples as 10 samples are not enough for relevant quantitative statistic evaluations which are important especially for brittle materials. This is demonstrated on the used cutting crowns in Figure 16 (right), where the lower one (Figure 16b) shows chipping at the edge resulting in failure and mass loss. On the other hand, additional work must be done according to the temperature distribution during heating and melting in the printing machine. Early failed samples with no clear projectile impact must be further investigated to understand if there are any microstructural changes that might affect wear resistance. 

## 4. Conclusions

The results demonstrate the feasibility of the PBF-EB technology for additive manufacturing of cutting crowns. In addition, near-net-shape and thin-walled structures based on FeCr-10V can be built up without tools for the first time as existing processes only produce thin coatings or require massive and cost-intensive post-processing. Of particular interest, here, is the wear resistance in the practical test, which is two to three times that of the reference materials. Further findings are:

1. No significant difference was found between thick-walled and thin-walled PBF-EB structures with the microstructural analysis methods used in this study.
2. EBSD and XRD revealed the main phases to be ferrite, VC, and Cr_7_C_3_. However, there was a significant fraction that could not be analyzed by the present measurements, indicating retained austenite, martensite, and small amounts of other carbides and imbalance phases.
3. The as-built microstructure is fine grained with the majority of grains and carbides being submicron in size. The heat treatment has an effect mainly on the matrix structure and the formation of chromium carbides. The extent to which the composition of the chromium and vanadium-rich carbides change, as well as their correlation with the hardness values must be investigated in greater depth in the future.
4. Vanadium-rich primary carbides partly form star-like agglomerates.
5. Bending strength of heat-treated FeCr-10V is higher than 2000 MPa. Further investigations on the heat treatment accompanied by detailed microstructural and fracture analyses might give information about the influence of carbide formation on the ductility.
6. The jet wear test resulted in a mass loss that was only 2/3 or even half that of the reference material. The reason, especially for the good durability in the as-built condition, is not yet understood. In the future, correlations among microstructure, beam angle, and the wear mechanisms at different angles must be further studied.
7. Drop energy of heat-treated FeC10V lay in the range of that of the reference material. Further measurements have to be done for statistical relevance.
8. Thermal properties are in accordance with the literature for cold work steels.

In the future, a more comprehensive characterization of the microstructures, in heat-treated state especially, will be required to further understand and optimize their properties fully. In addition, a larger number of cutting crowns and mechanical specimens must be tested in order to obtain statistically relevant values of the rather brittle materials.

The results give an idea of the new capabilities with these materials in case of near-net-shape structures. One of these options comprises the use of thin-walled PBF-EB parts for hot isostatic pressing (HIP) which addresses the following advantages:

1. Near-net-shape capsule for HIP using PBF-EB.
2. In situ hard facing by the FeCr-10V, with varying wall thicknesses depending on the application.
3. Core material of the capsule might be a more cost-effective material with higher toughness.
4. Hybrid manufacturing leads to reduced PBF-EB printing times that are still a bottleneck for industrial production.

Finally, the results serve as an indication of what can be achieved in terms of wear resistance of even higher VC-loaded and other MMC-containing alloys. The areas of application will become even more versatile if investigations are also carried out on corrosion-resistant austenitic Fe-VC alloys.

## Figures and Tables

**Table 1:** Heat-treating (QT) parameters for PBF-EB-processed FeCr-10V.

Austenitization	Quenching	Tempering
1020 °C (1 h)	oil (1020 °C → 500 °C)/ air (from 500 °C)	3 × 540 °C (1 h)

**Table 2:** Summary of the phases detected in as-built FeCr-10V by EBSD. Colors refer to Figure 7a.

Phase	Color	Total Fraction	Partition Fraction
bcc–ferrite (alpha iron)		0.511	0.648
fcc–mainly v-rich carbides		0.258	0.327
hex–M_7_C_3_ (Cr-rich)		0.020	0.025

## Data Availability

The data that support the findings of this study are available from the corresponding author upon reasonable request.
